# Are Competence Beliefs or Value Beliefs More Important for STEM Career Aspirations?—Longitudinal Mediation Analyses Based on Recent Modeling Approaches Show Different Results

**DOI:** 10.1007/s10964-025-02162-3

**Published:** 2025-03-04

**Authors:** Tobias Debatin, Heidrun Stoeger, Albert Ziegler

**Affiliations:** 1 Department of Educational Sciences, University of Regensburg, Regensburg, Germany; 2 Department of Educational Psychology, Friedrich-Alexander University Erlangen-Nuremberg, Nuremberg, Germany

**Keywords:** Academic self-concept, STEM, Competence beliefs, Value beliefs, Career aspirations

## Abstract

There is a consensus in situated expectancy-value theory research that value beliefs better predict career aspirations and choices than competence beliefs and thus should be the main target of interventions aimed to motivate youth for STEM (science, technology, engineering, math) careers. However, research on the longer-term causal effects of the two constructs and their indirect effects is missing. The latter is relevant since competence beliefs seem to influence value beliefs but less the other way around. The present study investigated such longer-term effects and the underlying indirect effects in a sample of 690 students from Germany (52.6% girls; *M* (T1) = 15.26 years, *SD* = 0.68) over three measurement points from the middle of Grade 9 to the middle of Grade 10. For these purposes, two recent models with improved properties for identifying causal effects, the random intercept cross-lagged panel model (RI-CLPM) and the dynamic panel model, as well as the traditional CLPM were applied. Final analyses were based on the RI-CLPM, and the results were compared to the traditional CLPM. Both models found the expected tendency of higher effects of STEM value beliefs on STEM career aspirations from one time point to the next. However, regarding the longer-term total effects, the analysis based on the RI-CLPM found a slight tendency for larger effects of competence beliefs, including an indirect effect of competence beliefs via value beliefs. These findings indicate that the competence beliefs of youth should not be underestimated in developing interventions for career aspirations and choices.

## Introduction

Career choices are very important decisions in life with many long-term consequences. In recent years, much attention has been paid to what motivates youth to pursue STEM (science, technology, engineering, mathematics) careers, given the high demand in the current labor market. The situated expectancy-value theory (Eccles & Wigfield, 2020; Wigfield & Eccles, 2000) is one of the most well-known theories for explaining how such occupational choices arise. The theory has the following simplified core assumption, which was confirmed by a large number of studies over the last decades (for a summary see Eccles & Wigfield, 2020): Individuals choose to engage in tasks, activities, and domains they expect to do well in (expectancies for success, or more generally competence beliefs, often operationalized via academic self-concepts) and in which they see value (value beliefs). The situated expectancy-value theory assumes additionally that value beliefs are stronger predictors of choices than competence-related variables (Eccles & Wigfield, 2024) and empirical support of this assumption led to the recommendation that value beliefs should be the main target of career choice interventions (for a recent review see Rosenzweig et al., 2022). However, research on the longer-term causal effects (assessed over more than two time points and with current models) of the two constructs and their indirect effects is missing. This study re-examines the empirical evidence of the special importance of value beliefs for career aspirations and choices in the STEM domain as it is highly relevant for planning interventions.

### Situated Expectancy-Value Theory and Open Questions Regarding Interventions

According to situated expectancy-value theory (Eccles & Wigfield, 2024), individuals choose to engage in tasks, activities, and domains in which they expect to do well, and which have a high overall subjective value for them. One’s overall value is assumed to be an aggregate of intrinsic value (a task or domain is enjoyable), attainment value (the importance of a task or domain), utility value (a task or domain is useful (for one’s plans)), and cost (the cost/benefit ratio of a task or domain). It is ingrained in situated expectancy-value theory that these value beliefs are more important for engagement and choices than competence beliefs, while competence beliefs are more important for achievement (Eccles & Wigfield, 2024; Rosenzweig et al., 2022). However, while the higher predictive strength of value beliefs for career choices is empirically well established in observational studies and interventions typically focus on value beliefs (for a recent review see Rosenzweig et al., 2022), from a developmental perspective, two related aspects complicate the decision whether to focus more on value beliefs or competence beliefs in interventions, especially when the interventions are conducted a relatively long time before the actual career choices.

First, besides the direct effects of competence beliefs and value beliefs on career aspirations and choices from one time point to the next, situated expectancy-value theory also assumes indirect effects over time. The most prominent one is the indirect effect of competence beliefs on career aspirations and choices via value beliefs. It is assumed that students start to value what they feel competent in as this is intrinsically rewarding, probably primarily due to eliciting positive affect, similar to processes in classical conditioning and positive reinforcement (Eccles, 2009). Additionally, students seem to maintain a positive view of themselves by devaluing tasks or domains in which they feel not competent (Eccles, 2009). Furthermore, an indirect effect from value beliefs on career aspirations and choices via competence beliefs is also expected as increased engagement in the domain, resulting from valuing it, can lead to higher skills and, in consequence, an increase in competence beliefs (Eccles, 2009). Empirical findings indicate that the development of value beliefs indeed depends on competence beliefs as assumed, but much less the other way around (for a summary, see Benden & Lauermann, 2023). This implies an indirect effect of competence beliefs on career aspirations and choices via value beliefs and only a smaller indirect effect from value beliefs via competence beliefs, if at all. Accordingly, value beliefs should mostly have a direct effect on career aspirations and choices, and the longer-term effect of increasing value beliefs at a certain time point should strongly depend on how much the consequent increase in career aspirations carries over to later points in time; technically this is also an indirect effect via earlier levels of career aspirations.

These considerations of indirect effects, which must be interpreted causally to make sense (e.g., MacKinnon & Pirlott, 2015; Rohrer et al., 2022), lead to the second point complicating the decision of what to focus on in interventions when based on observational data: Prediction is not causation and to evaluate what consequences changes in competence beliefs and value beliefs have, a strong emphasis should be placed on evidence for causality. However, aside from randomized trials, causal evidence is very hard to obtain as it needs special longitudinal designs and analyses to provide at least a certain degree of causal evidence (Murayama & Gfrörer, 2024; Vanderweele et al., 2020). In the domain of interest, STEM, there are surprisingly few longitudinal studies regarding both the mentioned indirect effects of competence beliefs and value beliefs on career aspirations and choices as well as regarding evidence for causality from observational data. Most previous situated expectancy-value theory studies, which found the discussed dominance of value beliefs, examined competence beliefs and value beliefs at a single point in time simultaneously and predicted outcomes such as STEM career aspirations (e.g., Jansen et al., 2021) or choices (e.g., Wille et al., 2020) at a single later point in time, controlling for basic covariates. It is reasonable to assume that only the inclusion of basic covariates is not sufficient to reduce confounding substantially as the outcome at an earlier time point is the most impactful covariate for reducing confounding (Vanderweele et al., 2020), acting as a confound-blocker (Wysocki et al., 2022). To be fair, when studies investigate future STEM choices like studying a STEM subject, there is, of course, no identical measure of the outcome at a previous time point to control for. The situation is different, however, in longitudinal studies examining the effects of competence beliefs and value beliefs on career aspirations. In this case the possibility exists to ask repeatedly about career aspirations and include the outcome at a previous time point in the model, which reduces the risk of confounding substantially. Still, there are only two such studies in the context of situated expectancy-value theory assessing competence beliefs, value beliefs, and STEM career aspirations at more than one time point (namely two) and controlling for the previous time point(s) (Lauermann et al., 2017; Lazarides & Lauermann, 2019). Both studies found the typical effects of competence beliefs (assessed via self-concepts) and value beliefs on career aspirations, but interestingly, these studies did not find stronger effects of value beliefs compared to competence beliefs, indicating that both constructs could be equally important targets for interventions to improve STEM career aspirations.

### Missing Longitudinal Mediation Analyses and Advantages of Recent Models

The above-mentioned studies assessing the main constructs of situated expectancy-value theory at two time points provide some evidence for effects involved in the assumed indirect effects (for example, the causal path from competence beliefs to the mediator value beliefs or the causal path from the mediator value beliefs to the outcome career aspirations). However, two time points are insufficient for properly testing complete mediation chains, for example, from self-concepts to value beliefs to career aspirations. The reason is that the proper temporal order cannot be ensured with only two time points. To investigate whether competence beliefs influence value beliefs and whether value beliefs influence career aspirations would need three time points, the only other option being to make more untestable assumptions (e.g., that effects are stable over time). Modeling over more than two time points allows to investigate how the system develops from state to state and if the effects between the variables are stable over time. Additionally, it allows to estimate total effects over the whole study period (Cole & Maxwell, 2003). These effects consist of all possible paths between two variables over the study period.

Accordingly, conducting studies with the relevant situated expectancy-value theory variables assessed at more than two time points and conducting a traditional longitudinal mediation analysis using the standard cross-lagged panel model (CLPM; Cole & Maxwell, 2003) would have advantages, but other problems remain. For example, in the very likely scenario that there are unmeasured confounding variables that affect both the outcome and the predictor directly (for example, rather time-invariant variables such as the socio-economic background), the results of traditional CLPMs become biased (see Murayama & Gfrörer (2024) for a detailed description which (other) biases can arise with the CLPM). For cross-lagged effects, such bias may sometimes be small enough not to distort results too much (Lüdtke & Robitzsch, 2023). However, for autoregressive effects, the bias can always be expected to be large as it is not possible for the outcome at a previous time point to be a confound blocker for itself. This means many variables could confound the autoregressive effect between the outcome and the outcome at a previous time point in the CLPM. Therefore, the autoregressive effect depends much more than the cross-lagged effect on additional covariates to reduce confounding. As it can be argued that the covariates assessed in typical studies are very often clearly not sufficient, CLPMs are usually not well suited for longitudinal mediation analyses, especially due to biased autoregressive effects. Fortunately, recent extensions of the CLPM like the random-intercept cross-lagged panel model (RI-CLPM; Hamaker et al., 2015) and the predetermined dynamic panel model (DPM; Allison et al., 2017; Andersen, 2022), can (better) account for unobserved confounding variables by including latent variables (Usami et al., 2019). These methods improve causal estimates for cross-lagged and autoregressive effects in many realistic scenarios and should be a clear improvement, especially for longitudinal mediation analyses. However, while including latent variables probably reduces the confounding of autoregressive effects substantially, it should be kept in mind that it still is an approximation that cannot substitute an analysis in which all relevant confounders are assessed directly.

## Current Study

While there is a consensus in research on situated expectancy-value theory that value beliefs better predict career aspirations than competence beliefs, it is less clear if this also applies to the longer-term causal effects of the two constructs involving their indirect effects. Indirect effects are relevant since competence beliefs seem to influence value beliefs but less the other way around. Accordingly, the present study aimed to fill this gap by conducting a longitudinal mediation analysis in the STEM domain with the main constructs of situated expectancy-value theory: competence beliefs (operationalized via academic self-concepts), value beliefs, and career aspirations. Direct positive causal effects of STEM self-concepts (Hypothesis 1) and STEM value beliefs (Hypothesis 2) on STEM career aspirations were expected from one time point to the next. The effects of STEM value beliefs were assumed to be larger than the effects of STEM self-concepts. Additionally, an indirect positive causal effect of STEM self-concepts on STEM career aspirations via STEM value beliefs (Hypothesis 3) and an indirect positive causal effect of STEM value beliefs on STEM career aspirations via STEM self-concepts (Hypothesis 4) were expected. This second indirect effect was expected to be smaller, mostly due to the findings that competence beliefs like academic self-concepts influence value beliefs, but much less the other way around. Finally, positive and causal total effects of STEM self-concepts (Hypothesis 5) and STEM value beliefs (Hypothesis 6) on STEM career aspirations were expected over the whole study period. Total effects are the addition of all possible paths between two variables at two time points (Cole & Maxwell, 2003), in the recent study between the first and the third time point, thereby involving the indirect effects. As larger direct effects of STEM value beliefs and a larger indirect effect of STEM competence beliefs via STEM value beliefs were expected, the effects were assumed to neutralize each other to a certain extent and lead to similar total effects.

## Method

### Sample and Procedure

The sample consists of 690 students (52.6% girls; *M* (T1) = 15.26 years, *SD* = 0.68) from 24 classes of nine “Gymnasien” (highest-level secondary school in the German tracking system). Students were in the middle of grade 9 at the beginning of the longitudinal study (T1 in April 2009), with two further assessments at the beginning of grade 10 (T2 in October 2009) and in the middle of grade 10 (T3 in March 2010). All students who participated at least at one of the three time points were included. Thirteen students who did not report their gender were excluded as we conducted gender invariance models and preferred having the same sample in each model. There were 33% missing value beliefs in the other variables (gender having no missing values) at the first time point, 34% missing values at the second time point, and 39% missing values at the third time point. The same data set has been used in two previous studies with completely different research questions (Debatin et al., 2023; Stoeger et al., 2016).

### Measures

#### STEM Self-Concept

Students’ STEM self-concepts were measured by adapting Dweck and Henderson’s (1988) scale of self-confidence in intelligence to STEM abilities. A 6-point Likert-type scale was used with two contrary statements as the endpoints for each item as in the original scale. Adapted item endpoints were as follows. Item 1: “I wonder if I am talented for the STEM subjects.” vs. “Overall, I think I am talented for the STEM subjects.”. Item 2: “When I get new learning material in the STEM subjects, I often think I may not be able to understand it” vs. “When I get new learning material in the STEM subjects, I am usually able to understand it.”. Item 3: “I am not very confident about my abilities for the STEM subjects.” vs. “I feel fully confident about my abilities for the STEM subjects.”. A fourth item was added: “I am not sure if I am good enough to succeed in the STEM subjects.” vs. “I am sure that I am good enough to succeed in the STEM subjects.”. While statements about being talented and understanding content are typical for self-concept items (e.g., Marsh et al., 2019, 2022), other wordings of the scale might also be classified as measuring generalized self-efficacy or success expectancies in STEM. However, it was found that measures of generalized self-efficacy as well as success expectancies are empirically close to indistinguishable from (academic) self-concept (Marsh et al., 2019). Cronbach’s alpha was 0.92, 0.94, and 0.94 for the three time points.

#### STEM Value Beliefs

STEM value beliefs were measured by an aggregate score of six 6-point Likert-type items assessing intrinsic value, attainment value, and utility value, with two items each, one related to “STEM subjects” and the other related to “STEM lessons” (Ziegler et al., 2008). Example items are: “I like the STEM lessons” (intrinsic value), “Being good at the STEM subjects is important to me” (attainment value), and “The STEM lessons can be put to good use” (utility value). Cronbach’s alpha was 0.86, 0.86, and 0.89 for the three time points.

#### STEM Career Aspirations

STEM career aspirations were measured by the two 6-point Likert-type items “I could imagine studying a STEM subject” and “I could see myself pursuing a career related to the STEM field.”. Cronbach’s alpha was 0.90, 0.92, and 0.92 for the three time points.

### Data Analysis

For the longitudinal mediation analyses, three models were calculated: The traditional CLPM (lag 1), the RI-CLPM, and a predetermined DPM (as described in Andersen, 2022), all of them with all possible paths (lag 1) between the three variables over the three time points. Next, the model fits of all three models were compared. As expected, and reported below, the traditional CLPM showed the worst model fit. However, as it is the traditional basis for longitudinal mediation analyses, we report and compare its results with the extended models.

The comparison between the (predetermined) DPM and the standard RI-CLPM should often be a default procedure as one common scenario in which the more parsimonious standard RI-CLPM should have more bias and worse model fit than the DPM (or a RI-CLPM with free covariances between the initial deviations and the random intercepts, which has an identical model fit as a predetermined DPM given time-invariant paths; Andersen, 2022) is if a process just started and/or is not (yet) at equilibrium, meaning stable means, variances, and covariances during the study period of observation (Andersen, 2022). In a sample of adolescents, it seemed at least possible that the process of developing career aspirations only started recently. DPM was the starting point and was compared to the model fit of the more parsimonious RI-CLPM. As reported below, the DPM showed no better model fit, and the RI-CLPM results were used as the basis for the longitudinal mediation analyses due to parsimony.

In the following, explanations are provided why mean scores were used instead of latent variables in the final models. As a first pragmatic reason, there is no research how to best deal with (within-person) correlated uniqueness in the latent version of the RI-CLPM (Mulder & Hamaker, 2021). Mean scores should be more robust concerning this problem (at least when indicators have different levels of correlated uniqueness) and regarding interpretational confounding (Rhemtulla et al., 2020). Additionally, while a reflective measurement model makes sense for STEM self-concept and STEM career aspirations, it does not for STEM value beliefs as there is no assumption of a latent variable which is the (single) cause of different kinds of STEM value beliefs. Therefore, mean scores should be used as otherwise distortions of the meaning and structural parameters would arise (Rhemtulla et al., 2020). As the (composite) mean scores of the STEM value beliefs contain measurement error, modeling STEM self-concepts and STEM career aspirations as latent variables would cause another problem: Using error-free variables as well as variables with measurement error in the same model, would probably lead to positively biased estimates of the paths of the latent variables and negatively biased paths of the variables with measurement error (Cole & Preacher, 2014). Considering that all mean score variables contain few measurement error (very high reliabilities between 0.86 and 0.94), the decision was that it is better to accept that all paths are probably slightly negatively biased when using mean scores (Cole & Preacher, 2014).

### Estimation of the Models

R 4.3.1 (R Core Team, 2023) with the lavaan package (v0.6-16; Rosseel, 2012) was used for the main analyses. The DataExplorer package (v0.8.2; Cui, 2020) was used to get the missing data profile and the distributions of the variables. The robust maximum likelihood estimator (MLR) with the cluster command of lavaan was used for all analyses to appropriately adjust the standard errors as students were clustered within classes. Model fit was assessed following the criteria of Hu and Bentler (1999). Therefore, a value close to 0.95 for the Comparative Fit Index (CFI), a value close to 0.06 for the root mean squared error of approximation (RMSEA), and a value close to 0.08 for the standardized root mean squared residual (SRMR) were the cutoff criteria for assuming good model fit. Missing values were handled using the full information maximum likelihood method (FIML), which is especially suitable for longitudinal studies assessing the same variables at different time points. In this case, no further auxiliary variables should be necessary (Graham, 2009).

The analysis code for the main analyses of this study can be found in the online supplemental material. Original data are not publicly available but can be requested by emailing the corresponding author.

## Results

Following the recommendations in a recent editorial of the executive director of the American Statistical Association (ASA) and colleagues (Wasserstein et al., 2019), this manuscript does not use the term “statistically significant” or a particular threshold for p values like <0.05. Instead, *p* values are treated “as a continuous measure of the compatibility between the data and the entire model used to compute it”, as supported by the ASA (Greenland et al., 2016, p. 339). The term “entire model” is used to highlight the fact that *p* values do not solely depend on how compatible the data are with a test hypothesis (often a null hypothesis). Instead, violations of a host of other assumptions about data generation, such as a properly conducted analysis, proper measurement of constructs, and adherence to the study protocols, may impact *p* values (Amrhein et al., 2019; Greenland et al., 2016). In other words, any violation of the aforementioned assumptions could lead to low or high *p* values. Accordingly, the point estimates of the effects are the effect sizes most compatible with the data (they have *p* = 1) if all other assumptions are correct. Evidence for or against hypotheses is judged by considering a combination of precise *p* values (except when *p* < 0.001), effect sizes, standard errors, consistency with theory and prior results, and factors like measurement quality. Table 1 shows the descriptive statistics and correlations for all variables.

**Table 1:** Descriptive statistics and correlations for all variables used in the final models

	1	2	3	4	5	6	7	8	9	10
STEM self-concept										
1. T1	—									
2. T2	0.77	—								
3. T3	0.74	0.77	—							
STEM value beliefs										
4. T1	0.56	0.54	0.50	—						
5. T2	0.52	0.55	0.53	0.69	—					
6. T3	0.41	0.47	0.51	0.64	0.72	—				
STEM career asp.										
7. T1	0.61	0.60	0.55	0.58	0.47	0.40	—			
8. T2	0.56	0.62	0.59	0.55	0.61	0.48	0.76	—		
9. T3	0.52	0.59	0.61	0.52	0.60	0.61	0.69	0.80	—	
Female gender	−0.31	−0.35	−0.32	−0.16	−0.17	−0.14	−0.30	−0.29	−0.24	—
Descriptives										
*M*	3.70	3.68	3.77	4.47	4.48	4.39	3.65	3.57	3.59	53%
*SD*	1.30	1.31	1.32	0.87	0.80	0.86	1.47	1.50	1.53	—

*N* = 690. All correlations have *p* < 0.05. All variables other than gender (0 = male, 1 = female) ranged from 1 to 6, with 6 indicating the highest

### Gender Invariance and Model Fit Comparisons for Selecting the Final Models

For the CLPM and the RI-CLPM multi-group versions with invariant paths between genders and invariant paths over time showed no evidence of worse model fit than models without these constraints (see online supplemental materials for all multi-group model fit comparisons). The standard RI-CLPM with these group and time invariance constraints also showed no evidence of worse model fit than the predetermined DPM with these constraints (or a RI-CLPM with free covariances between the initial deviations and the random intercepts, which has an identical model fit as a predetermined DPM given time-invariant paths; Andersen, 2022). As the standard RI-CLPM is the more parsimonious model compared to the predetermined DPM it was decided to use the standard RI-CLPM with invariant paths over time for the longitudinal mediation analyses and to compare the mediation results based on this model with the traditional results of the CLPM with invariant paths over time.

In line with expectations, model fit in the traditional CLPM (χ^2^(18) = 79.17, *p* < 0.001; CFI(robust) = 0.97, RMSEA(robust) = 0.12, SRMR = 0.03) was considerably worse compared to the RI-CLPM including random intercepts for each variable (χ^2^(12) = 12.52, *p* = 0.405; CFI(robust) = 1.00, RMSEA(robust) = 0.01, SRMR = 0.02). The Satorra-Bentler Scaled Chi-Square Difference Test clearly confirmed the results regarding better model fit of the RI-CLPM (χ^2^diff(6) = 46.96, *p* < 0.001).

### Hypotheses 1 and 2: Direct Effects of STEM Self-Concepts and STEM Value Beliefs

As supported by the American Statistical Association, one-sided *p* values are used for the one-sided hypotheses (point 14 in Greenland et al., 2016). As reported in Table 2, there was evidence for the assumed direct positive effects of STEM self-concepts and STEM value beliefs on STEM career aspirations in the traditional CLPM and the RI-CLPM. The expected tendency of higher effects of STEM value beliefs was also present. Effect sizes were generally larger in the RI-CLPM, and *p*-values and standard errors were higher. Complete results, also of all other direct (lag 1) effects, can be found in Table 2. More detailed results, including unstandardized effects, can be found in the online supplemental materials.

**Table 2:** Standardized path coefficients of the CLPM & RI-CLPM

	CLPM			RI-CLPM		
Paths T1 → T2	Est.	*SE*	*p*	Est.	*SE*	*p*
STEM career aspirations → STEM career aspirations	0.62	0.04	<0.001	0.33	0.07	<0.001
STEM self-concepts → STEM career aspirations	0.10	0.03	<0.001	0.15	0.07	0.019
STEM value beliefs → STEM career aspirations	0.15	0.04	0.001	0.20	0.11	0.037
STEM value beliefs → STEM value beliefs	0.63	0.04	<0.001	0.09	0.16	0.296
STEM self-concepts → STEM value beliefs	0.14	0.04	<0.001	0.25	0.08	<0.001
STEM career aspirations → STEM value beliefs	0.02	0.03	0.383	0.11	0.12	0.367
STEM self-concepts → STEM self-concepts	0.63	0.04	<0.001	0.11	0.12	0.173
STEM value beliefs → STEM self-concepts	0.10	0.04	0.006	0.20	0.11	0.036
STEM career aspirations → STEM self-concepts	0.14	0.03	<0.001	0.25	0.09	0.008

Paths T2 → T3	Est.	*SE*	*p*	Est.	*SE*	*p*
STEM career aspirations → STEM career aspirations	0.65	0.04	<0.001	0.38	0.08	<0.001
STEM self-concepts → STEM career aspirations	0.11	0.03	0.001	0.17	0.08	0.020
STEM value beliefs → STEM career aspirations	0.14	0.04	0.001	0.17	0.11	0.067
STEM value beliefs → STEM value beliefs	0.60	0.04	<0.001	0.06	0.13	0.318
STEM self-concepts → STEM value beliefs	0.14	0.04	<0.001	0.23	0.09	0.004
STEM career aspirations → STEM value beliefs	0.02	0.03	0.396	0.11	0.13	0.415
STEM self-concepts → STEM self-concepts	0.64	0.03	<0.001	0.11	0.13	0.188
STEM value beliefs → STEM self-concepts	0.09	0.04	0.007	0.16	0.10	0.059
STEM career aspirations → STEM self-concepts	0.15	0.03	<0.001	0.28	0.09	0.002

*N* = 690. *P* value beliefs are one-sided due to our one-sided hypotheses, except for the paths from STEM career aspirations to STEM self-concepts and STEM value beliefs, as we had no clear hypotheses, and thus, two-sided *p* value beliefs are more appropriate

### Hypotheses 3 and 4: Indirect Effects of STEM Self-Concepts and STEM Value Beliefs

There was evidence for the assumed indirect positive effect of STEM-self-concepts (T1) on STEM career aspirations (T3) via STEM value beliefs (T2) in the traditional CLPM (standardized indirect effect: 0.02, *p* = 0.005, one-sided, 95% CI [0.01, 0.04]) as well as in the RI-CLPM (standardized indirect effect: 0.05, *p* = 0.052, one-sided, 95% CI [−0.01, 0.11]). There was also evidence for the assumed indirect positive effect of STEM value beliefs (T1) on STEM career aspirations (T3) via STEM self-concepts (T2) in the traditional CLPM (standardized indirect effect: 0.01, *p* = 0.011, one-sided, 95% CI [0.00, 0.02]) as well as in the RI-CLPM (standardized indirect effect: 0.03, *p* = 0.115, one-sided, 95% CI [−0.02, 0.08]), though as expected to a lesser degree. More detailed results, including unstandardized effects, can be found in the online supplemental materials.

### Hypotheses 6 and 7: Total Effects of STEM Self-Concepts and STEM Value Beliefs

There was evidence for the assumed positive total effect of STEM self-concept (T1) on STEM career aspirations (T3) in the traditional CLPM (standardized total effect: 0.15, *p* < 0.001, one-sided, 95% CI [0.08, 0.21]) as well as in the RI-CLPM (standardized total effect: 0.12, *p* = 0.008, one-sided, 95% CI [0.02, 0.21]). There was also evidence for the assumed positive total effect of STEM value beliefs (T1) on STEM career aspirations (T3) in the traditional CLPM (standardized total effect: 0.19, *p* < 0.001, one-sided, 95% CI [0.09, 0.29]) as well as in the RI-CLPM (standardized total effect: 0.11, *p* = 0.079, one-sided, 95% CI [−0.04, 0.27]). As expected, the effect sizes were similar, at least in the RI-CLPM. However, evidence for the total effect of STEM competence beliefs was stronger than for STEM value beliefs in the RI-CLPM, as indicated by a much lower *p* value. More detailed results, including unstandardized effects, can be found in the online supplemental materials.

## Discussion

Research on situated expectancy-value theory agrees that value beliefs better predict career aspirations and choices than competence beliefs (Rosenzweig et al., 2022). However, it is less clear if this also applies to the longer-term causal effects of the two constructs involving their indirect effects, especially since competence beliefs seem to influence value beliefs but less the other way around. The present study analyzed and compared the influence of STEM competence beliefs (assessed via STEM self-concepts) and STEM value beliefs over three points in time with a longitudinal mediation analysis. This filled the important research gap regarding longer-term causal effects and the involved indirect effects, as there is no complete longitudinal mediation analysis to date that covers the required three points in time and allows for adequate control of confounding. The final analysis was based on the RI-CLPM, which has improved properties for identifying causal effects from longitudinal data (in many realistic scenarios). Results were compared to a traditional autoregressive mediation analysis based on the CLPM. Both the traditional analysis and the analysis based on the RI-CLPM confirmed prior findings of a stronger direct effect of STEM value beliefs on STEM career aspirations from one time point to the next. However, the results differed regarding the (longer-term) total effects of changes of the two variables at the beginning of the study period on STEM career aspirations at the end of the study period, which involve several indirect paths. The traditional analysis found a tendency for a larger total effect of STEM value beliefs; the analysis based on the RI-CLPM found no differences with a slight tendency for a larger total effect of competence beliefs. Additionally, the total effects based on the RI-CLPM were generally lower.

### Direct Effects of Self-Concepts and Value Beliefs on Career Aspirations in STEM

There was evidence for the assumed direct positive effects of STEM self-concepts and STEM value beliefs on STEM career aspirations from one time point to the next in the CLPM and the RI-CLPM. The expected tendency of higher effects of STEM value beliefs was also present. While the effect size differences weren’t large, especially in the RI-CLPM, overall the results are consistent with the common assumption that the effects of value beliefs are larger, at least from one point in time to the next. The most striking difference between the results of the two models regarding direct effects from one time point to the next is the difference in the autoregressive effect sizes with much lower effects in the RI-CLPM. Lower autoregressive effects than in the CLPM are common when applying the RI-CLPM (Ehm et al., 2021; Marsh et al., 2022; Mulder & Hamaker, 2021). As they should be a much less biased estimate than the autoregressive effects from the CLPM, this indicates that the effects of competence beliefs and value beliefs on career aspirations, and more generally every change in career aspirations seem to decay much faster than the relatively large estimates from the CLPM suggest. Such an overestimation of traditional methods was also found and validated in research on skill building (Bailey et al., 2018). Possibly, more enduring interventions like mentoring could be needed to better induce long-term changes (Stoeger et al., 2023).

### Indirect effects of Self-Concepts and Value Beliefs on Career Aspirations in STEM

In both models, there was evidence for the assumed indirect positive effect of STEM-self-concepts (T1) on STEM career aspirations (T3) via STEM value beliefs (T2). There was also evidence in both models for the assumed indirect positive effect of STEM value beliefs (T1) on STEM career aspirations (T3) via STEM self-concepts (T2), though as expected to a lesser degree (i.e., lower effect sizes and higher *p* values). While this finding that the indirect effect of STEM self-concepts via value beliefs is larger is in line with previous research (Benden & Lauermann, 2023), an explanation could be based on a basic need for competence (Ryan & Deci, 2000), the fulfillment of which can be intrinsically rewarding over time, leading to (intrinsically) valuing the corresponding domain (Eccles, 2009). At this point, it should be highlighted that the time interval from one point in time to the next was six months, and accordingly, the indirect effects refer to a period of twelve months. It seems likely that these indirect effects also occur within six months, meaning the “direct” effects mentioned in the previous section partially consist of such indirect effects, and therefore, the indirect effects reported here are probably underestimated (Cole & Maxwell, 2003).

### Total effects of Self-Concepts and Value Beliefs on Career Aspirations in STEM

As mentioned at the beginning of the discussion, there was also evidence in both models for the assumed positive total effects of STEM self-concepts (T1) on STEM career aspirations (T3) and of STEM value beliefs (T1) on STEM career aspirations (T3). Interestingly, only in the traditional analysis was the total effect of STEM value beliefs higher than the total effect of STEM self-concepts, and the results based on the RI-CLPM were generally lower. These important differences are due to the very different effect sizes of the autoregressive effects, which were at least about two times higher in the CLPM, some even about ten times higher. As the much lower autoregressive effects in the RI-CLPM should be substantially less biased estimates for causal effects and are an important part of the total effects, the total effects from the CLPM are not trustworthy. The lower total effect sizes due to lower autoregressive effects imply that over more extended periods (one year in the current study), the effects of changes in competence beliefs and value beliefs on career aspirations are smaller (decaying more quickly) than indicated by traditional CLPM analyses. Additionally, especially the effect of value beliefs seems to be lower than assumed based on traditional methods, also due to the relatively small indirect effect via self-concepts.

### Limitations

While the study extends previous findings in important ways, it also has limitations. First, due to the higher model complexity and the typically larger standard errors in the RI-CLPM and similar methods like the DPM compared to the CLPM (Mulder & Hamaker, 2021), a larger sample size or more time points would have been preferable to improve the strength of evidence derived from the RI-CLPM and the DPM. Second, many assumptions are involved in causal inference (e.g., Rubin, 2005), an important one being that all relevant confounders are appropriately considered in the analysis. This assumption alone is close to impossible to fulfill, also in the present study where many of the most important confounders, especially for the autoregressive effects, should be time-invariant; a situation which the RI-CLPM should typically be able to tackle well. Additionally, time-varying confounders are possible but were not measured. Nonetheless, the RI-CLPM should provide clearly improved estimates compared to the ones from the CLPM, especially regarding the autoregressive effects. Future studies could compare results with models with a more comprehensive assessment of confounders. Finally, only self-concepts and value beliefs in the domain of STEM were used in the models. However, in line with the assumed importance of the intraindividual hierarchy of competence beliefs and value beliefs for occupational choices in expectancy-value theory (Eccles, 1994) and dimensional comparison theory (Möller & Marsh, 2013), research points to incremental effects of competence beliefs in different domains (Umarji et al., 2018) and value beliefs in different domains (Umarji et al., 2023) for predicting career choices. Accordingly, future research should extend the analyses by including competence beliefs and value beliefs from other domains like languages.

## Conclusion

There is a consensus in situated expectancy-value theory research that value beliefs better predict career aspirations and choices than competence beliefs and thus should be the main target of interventions aimed to motivate youth for STEM careers. However, research on the longer-term causal effects of the two constructs and their indirect effects is missing. The results of the present study imply that the higher predictive strength of STEM value beliefs regarding later STEM career aspirations and choices from one point in time to the next—which was mostly in line with the results in this study—does not automatically translate to a higher causal total effect estimate over several time points. This is also due to the stronger indirect effect of STEM competence beliefs via STEM value beliefs. Accordingly, this contradicts the typical assumptions that an intervention on STEM value beliefs would generally increase subsequent STEM career aspirations and decisions more than an intervention on STEM competence beliefs. Possibly, the best way of motivating youth for STEM careers in the long term is via interventions on competence beliefs, while the typical (utility) value interventions are better suited for more rapid effects. With sufficient time and resources, both competence beliefs and value beliefs should be targeted to maximize effectiveness. Additionally, the results imply that the effects of both competence beliefs and value beliefs on career aspirations over time are smaller and decay more quickly than assumed by traditional methods. This implies that one-time interventions might not be enough, and more enduring interventions like mentoring could be needed to induce long-term changes. Overall, we conclude that competency beliefs deserve more attention in interventions designed to motivate youth to pursue a STEM career.

## Supplementary information

Supplementary Material - Mediation STEM career aspiration
