# Supplementary material for: Are Competence Beliefs or Value Beliefs More Important for STEM Career Aspirations?—Longitudinal Mediation Analyses Based on Recent Modeling Approaches Show Different Results
Source: J Youth Adolesc. 2025 Mar 4;54(7):1768–78. doi: 10.1007/s10964-025-02162-3 (PMC12245932; doi:10.1007/s10964-025-02162-3)
Supplement: Supplementary file 1 — Supplementary Material - Mediation STEM career aspiration [file 10964_2025_2162_MOESM1_ESM.docx]

**Are Competence Beliefs or Value Beliefs More Important for STEM Career Aspirations?—Longitudinal Mediation Analyses Based on Recent Modeling Approaches Show Different Results**

**Supplementary Materials**

Note: cxmean2=career aspirations, sxmean=self-concepts, ixmean6=value beliefs

**Code final CLPM**

clpm_final <- '

c2mean2 ~ c*c1mean2+sc*s1mean+ic*i1mean6

c3mean2 ~ c*c2mean2+sc*s2mean+ic*i2mean6

s2mean ~ cs*c1mean2+s*s1mean+is*i1mean6

s3mean ~ cs*c2mean2+s*s2mean+is*i2mean6

i2mean6 ~ ci*c1mean2+si*s1mean+i*i1mean6

i3mean6 ~ ci*c2mean2+si*s2mean+i*i2mean6

# Estimate the covariance between the observed variables at the first wave

c1mean2 ~~ s1mean + i1mean6

s1mean~~i1mean6

# Estimate the covariances between the residuals of the observed variables

c2mean2 ~~ s2mean + i2mean6

s2mean~~i2mean6

c3mean2 ~~ s3mean + i3mean6

s3mean~~i3mean6

#indirect and total effects

sa1 := si*ic

sa2 := s*sc

sa3 := sc*c

totalsc := sa1+sa2+sa3

ia1 := is*sc

ia2 := i*ic

ia3 := ic*c

totalic := ia1+ia2+ia3

'

clpm_final_fit <- sem(clpm_final, data = cmdatalong2, missing='fiml', estimator='MLR', cluster = 'classID_num')

summary(clpm_final_fit, fit.measures = T, standardized = T, ci=T

**Code final RI-CLPM**

RICLPM_final <- '

#between part

RIc =~ 1*c1mean2 + 1*c2mean2 + 1*c3mean2

RIs =~ 1*s1mean + 1*s2mean + 1*s3mean

RIi =~ 1*i1mean6 + 1*i2mean6 + 1*i3mean6

#within part

wc1 =~ 1*c1mean2

wc2 =~ 1*c2mean2

wc3 =~ 1*c3mean2

ws1 =~ 1*s1mean

ws2 =~ 1*s2mean

ws3 =~ 1*s3mean

wi1 =~ 1*i1mean6

wi2 =~ 1*i2mean6

wi3 =~ 1*i3mean6

c1mean2 ~~ 0*c1mean2

s1mean ~~ 0*s1mean

i1mean6 ~~ 0*i1mean6

c2mean2 ~~ 0*c2mean2

s2mean ~~ 0*s2mean

i2mean6 ~~ 0*i2mean6

c3mean2 ~~ 0*c3mean2

s3mean ~~ 0*s3mean

i3mean6 ~~ 0*i3mean6

# Estimate the lagged effects between the variables

wc2 ~ c*wc1+sc*ws1+ic*wi1

wc3 ~ c*wc2+sc*ws2+ic*wi2

ws2 ~ cs*wc1+s*ws1+is*wi1

ws3 ~ cs*wc2+s*ws2+is*wi2

wi2 ~ ci*wc1+si*ws1+i*wi1

wi3 ~ ci*wc2+si*ws2+i*wi2

# Estimate the covariance between the variables at the first wave

wc1 ~~ ws1 + wi1

ws1~~wi1

# Estimate the covariances between the residuals of the variables

wc2 ~~ ws2 + wi2

ws2~~wi2

wc3 ~~ ws3 + wi3

ws3~~wi3

# Set correlations between the between-factors (random intercepts) and within-factors at wave 1 at 0

RIc + RIs + RIi ~~ 0*wc1 + 0*ws1 + 0*wi1

#indirect and total effects

sa1 := si*ic

sa2 := s*sc

sa3 := sc*c

totalsc := sa1+sa2+sa3

ia1 := is*sc

ia2 := i*ic

ia3 := ic*c

totalic := ia1+ia2+ia3

'

note: correlations between RIs and the within-factors at wave 1 makes it equivalent to a two-sided predetermined dynamic panel model as explained in Andersen (2022)

fitRICLPM_final <- sem(RICLPM_final, data = cmdatalong2, missing='fiml', estimator='MLR', cluster = 'classID_num')

summary(fitRICLPM_final, fit.measures = T, standardized = T, ci=T)

**Code final predetermined DPM**

DPM_final <- '

RIs =~ 1*s2mean + 1*s3mean

RIi =~ 1*i2mean6 + 1*i3mean6

RIc =~ 1*c2mean2 + 1*c3mean2

# Estimate the lagged effects between the variables

c2mean2 ~ c*c1mean2+sc*s1mean+ic*i1mean6

c3mean2 ~ c*c2mean2+sc*s2mean+ic*i2mean6

s2mean ~ cs*c1mean2+s*s1mean+is*i1mean6

s3mean ~ cs*c2mean2+s*s2mean+is*i2mean6

i2mean6 ~ ci*c1mean2+si*s1mean+i*i1mean6

i3mean6 ~ ci*c2mean2+si*s2mean+i*i2mean6

# Estimate the covariance between the variables at the first wave

c1mean2 ~~ s1mean + i1mean6

s1mean~~i1mean6

# Estimate the covariances between the residuals of the variables

c2mean2 ~~ s2mean + i2mean6

s2mean~~i2mean6

c3mean2 ~~ s3mean+i3mean6

s3mean~~i3mean6

#correlations between factors and variables at the first wave

s1mean ~~ RIs + RIi + RIc

i1mean6 ~~ RIs + RIi + RIc

c1mean2~~ RIs + RIi + RIc

'

fitDPM_final <- sem(DPM_final, data = cmdatalong2, missing='fiml', estimator='MLR', cluster = 'classID_num')

summary(fitDPM_final, fit.measures = T, standardized = T, ci=T)

**Multi-group (gender) model fit comparisons**

tv=time- and group-varying coefficients

gi=only group invariant coefficients

tgi= time- and group-invariant coefficients

**CLPM**

Scaled Chi-Squared Difference Test (method = “satorra.bentler.2001”)

lavaan NOTE:

The “Chisq” column contains standard test statistics, not the

robust test that should be reported per model. A robust difference

test is a function of two standard (not robust) statistics.

Df AIC BIC Chisq Chisq diff Df diff Pr(>Chisq)

clpm_tv 18 10398 10806 78.951

clpm_gi 36 10380 10706 96.525 14.8265 18 0.6738

clpm_tgi 45 10372 10658 107.202 8.7836 9 0.4575

**RI-CLPM**

Scaled Chi-Squared Difference Test (method = “satorra.bentler.2001”)

lavaan NOTE:

The “Chisq” column contains standard test statistics, not the

robust test that should be reported per model. A robust difference

test is a function of two standard (not robust) statistics.

Df AIC BIC Chisq Chisq diff Df diff Pr(>Chisq)

RICLPM_tv 6 10350 10812 6.7312

RICLPM_gi 24 10331 10712 23.4968 17.4838 18 0.4901

RICLPM_tgi 33 10320 10660 31.2190 5.8466 9 0.7552

**Comparison final DPM vs. final RI-CLPM**

Scaled Chi-Squared Difference Test (method = “satorra.bentler.2001”)

lavaan NOTE:

The “Chisq” column contains standard test statistics, not the

robust test that should be reported per model. A robust difference

test is a function of two standard (not robust) statistics.

Df AIC BIC Chisq Chisq diff Df diff Pr(>Chisq)

DPM_final 3 10416 10647 1.9748

RICLPM_final 12 10409 10599 12.5239 10.475 9 0.3134

**Results final CLPM**

Regressions:

Estimate Std.Err z-value P(>|z|) ci.lower ci.upper Std.lv Std.all

c2mean2 ~

c1mean2 (c) 0.648 0.035 18.709 0.000 0.580 0.716 0.648 0.621

s1mean (sc) 0.123 0.035 3.475 0.001 0.053 0.192 0.123 0.103

i1mean6 (ic) 0.257 0.075 3.424 0.001 0.110 0.404 0.257 0.146

c3mean2 ~

c2mean2 (c) 0.648 0.035 18.709 0.000 0.580 0.716 0.648 0.652

s2mean (sc) 0.123 0.035 3.475 0.001 0.053 0.192 0.123 0.106

i2mean6 (ic) 0.257 0.075 3.424 0.001 0.110 0.404 0.257 0.140

s2mean ~

c1mean2 (cs) 0.129 0.024 5.390 0.000 0.082 0.175 0.129 0.144

s1mean (s) 0.638 0.038 16.623 0.000 0.563 0.713 0.638 0.628

i1mean6 (is) 0.149 0.060 2.504 0.012 0.032 0.266 0.149 0.099

s3mean ~

c2mean2 (cs) 0.129 0.024 5.390 0.000 0.082 0.175 0.129 0.149

s2mean (s) 0.638 0.038 16.623 0.000 0.563 0.713 0.638 0.635

i2mean6 (is) 0.149 0.060 2.504 0.012 0.032 0.266 0.149 0.094

i2mean6 ~

c1mean2 (ci) 0.013 0.015 0.857 0.391 -0.017 0.042 0.013 0.023

s1mean (si) 0.089 0.024 3.666 0.000 0.041 0.136 0.089 0.138

i1mean6 (i) 0.604 0.031 19.359 0.000 0.543 0.665 0.604 0.630

i3mean6 ~

c2mean2 (ci) 0.013 0.015 0.857 0.391 -0.017 0.042 0.013 0.024

s2mean (si) 0.089 0.024 3.666 0.000 0.041 0.136 0.089 0.140

i2mean6 (i) 0.604 0.031 19.359 0.000 0.543 0.665 0.604 0.603

Covariances:

Estimate Std.Err z-value P(>|z|) ci.lower ci.upper Std.lv Std.all

c1mean2 ~~

s1mean 1.166 0.072 16.299 0.000 1.025 1.306 1.166 0.615

i1mean6 0.735 0.057 12.967 0.000 0.624 0.846 0.735 0.577

s1mean ~~

i1mean6 0.629 0.056 11.254 0.000 0.520 0.739 0.629 0.561

.c2mean2 ~~

.s2mean 0.197 0.040 4.970 0.000 0.120 0.275 0.197 0.258

.i2mean6 0.203 0.042 4.873 0.000 0.121 0.284 0.203 0.376

.s2mean ~~

.i2mean6 0.094 0.039 2.392 0.017 0.017 0.170 0.094 0.203

.c3mean2 ~~

.s3mean 0.137 0.032 4.250 0.000 0.074 0.200 0.137 0.195

.i3mean6 0.195 0.049 3.967 0.000 0.099 0.291 0.195 0.375

.s3mean ~~

.i3mean6 0.091 0.035 2.588 0.010 0.022 0.161 0.091 0.194

Intercepts:

Estimate Std.Err z-value P(>|z|) ci.lower ci.upper Std.lv Std.all

.c2mean2 -0.396 0.231 -1.716 0.086 -0.849 0.056 -0.396 -0.259

.c3mean2 -0.326 0.217 -1.502 0.133 -0.753 0.100 -0.326 -0.214

.s2mean 0.182 0.197 0.926 0.355 -0.203 0.568 0.182 0.139

.s3mean 0.313 0.187 1.676 0.094 -0.053 0.679 0.313 0.237

.i2mean6 1.399 0.123 11.351 0.000 1.157 1.640 1.399 1.680

.i3mean6 1.310 0.108 12.152 0.000 1.099 1.522 1.310 1.571

c1mean2 3.645 0.092 39.763 0.000 3.465 3.825 3.645 2.485

s1mean 3.693 0.067 54.851 0.000 3.561 3.825 3.693 2.858

i1mean6 4.472 0.045 98.707 0.000 4.384 4.561 4.472 5.152

Variances:

Estimate Std.Err z-value P(>|z|) ci.lower ci.upper Std.lv Std.all

.c2mean2 0.898 0.108 8.338 0.000 0.687 1.109 0.898 0.383

.c3mean2 0.769 0.074 10.379 0.000 0.624 0.915 0.769 0.332

.s2mean 0.654 0.080 8.182 0.000 0.497 0.810 0.654 0.379

.s3mean 0.638 0.069 9.249 0.000 0.503 0.773 0.638 0.366

.i2mean6 0.323 0.033 9.707 0.000 0.258 0.389 0.323 0.466

.i3mean6 0.350 0.059 5.989 0.000 0.236 0.465 0.350 0.504

c1mean2 2.152 0.088 24.440 0.000 1.979 2.324 2.152 1.000

s1mean 1.670 0.088 18.977 0.000 1.498 1.843 1.670 1.000

i1mean6 0.754 0.060 12.463 0.000 0.635 0.872 0.754 1.000

Defined Parameters:

Estimate Std.Err z-value P(>|z|) ci.lower ci.upper Std.lv Std.all

sa1 0.023 0.009 2.569 0.010 0.005 0.040 0.023 0.020

sa2 0.078 0.025 3.191 0.001 0.030 0.126 0.078 0.065

sa3 0.079 0.023 3.460 0.001 0.034 0.124 0.079 0.064

totalsc 0.180 0.044 4.112 0.000 0.094 0.266 0.180 0.149

ia1 0.018 0.007 2.458 0.014 0.004 0.033 0.018 0.010

ia2 0.155 0.047 3.326 0.001 0.064 0.247 0.155 0.092

ia3 0.166 0.043 3.863 0.000 0.082 0.251 0.166 0.090

totalic 0.340 0.088 3.879 0.000 0.168 0.511 0.340 0.192

**Results final RI-CLPM**

Regressions:

Estimate Std.Err z-value P(>|z|) ci.lower ci.upper Std.lv Std.all

wc2 ~

wc1 (c) 0.366 0.074 4.950 0.000 0.221 0.511 0.332 0.332

ws1 (sc) 0.224 0.110 2.034 0.042 0.008 0.439 0.154 0.154

wi1 (ic) 0.365 0.199 1.835 0.066 -0.025 0.755 0.200 0.200

wc3 ~

wc2 (c) 0.366 0.074 4.950 0.000 0.221 0.511 0.380 0.380

ws2 (sc) 0.224 0.110 2.034 0.042 0.008 0.439 0.168 0.168

wi2 (ic) 0.365 0.199 1.835 0.066 -0.025 0.755 0.169 0.169

ws2 ~

wc1 (cs) 0.200 0.067 2.965 0.003 0.068 0.332 0.251 0.251

ws1 (s) 0.114 0.123 0.920 0.357 -0.128 0.356 0.108 0.108

wi1 (is) 0.264 0.131 2.012 0.044 0.007 0.521 0.200 0.200

ws3 ~

wc2 (cs) 0.200 0.067 2.965 0.003 0.068 0.332 0.276 0.276

ws2 (s) 0.114 0.123 0.920 0.357 -0.128 0.356 0.113 0.113

wi2 (is) 0.264 0.131 2.012 0.044 0.007 0.521 0.162 0.162

wi2 ~

wc1 (ci) 0.054 0.064 0.835 0.404 -0.073 0.180 0.109 0.109

ws1 (si) 0.163 0.066 2.452 0.014 0.033 0.293 0.251 0.251

wi1 (i) 0.070 0.137 0.509 0.611 -0.198 0.338 0.086 0.086

wi3 ~

wc2 (ci) 0.054 0.064 0.835 0.404 -0.073 0.180 0.106 0.106

ws2 (si) 0.163 0.066 2.452 0.014 0.033 0.293 0.230 0.230

wi2 (i) 0.070 0.137 0.509 0.611 -0.198 0.338 0.061 0.061

Covariances:

Estimate Std.Err z-value P(>|z|) ci.lower ci.upper Std.lv Std.all

wc1 ~~

ws1 0.302 0.133 2.272 0.023 0.041 0.562 0.430 0.430

wi1 0.268 0.115 2.334 0.020 0.043 0.493 0.479 0.479

ws1 ~~

wi1 0.172 0.079 2.168 0.030 0.017 0.327 0.405 0.405

.wc2 ~~

.ws2 0.228 0.060 3.786 0.000 0.110 0.346 0.377 0.377

.wi2 0.194 0.072 2.700 0.007 0.053 0.334 0.497 0.497

.ws2 ~~

.wi2 0.117 0.055 2.150 0.032 0.010 0.224 0.389 0.389

.wc3 ~~

.ws3 0.151 0.049 3.075 0.002 0.055 0.247 0.273 0.273

.wi3 0.191 0.047 4.026 0.000 0.098 0.283 0.460 0.460

.ws3 ~~

.wi3 0.096 0.043 2.232 0.026 0.012 0.181 0.277 0.277

RIc ~~

wc1 0.000 0.000 0.000 0.000 0.000

ws1 0.000 0.000 0.000 0.000 0.000

wi1 0.000 0.000 0.000 0.000 0.000

RIs ~~

wc1 0.000 0.000 0.000 0.000 0.000

ws1 0.000 0.000 0.000 0.000 0.000

wi1 0.000 0.000 0.000 0.000 0.000

RIi ~~

wc1 0.000 0.000 0.000 0.000 0.000

ws1 0.000 0.000 0.000 0.000 0.000

wi1 0.000 0.000 0.000 0.000 0.000

RIc ~~

RIs 0.850 0.144 5.910 0.000 0.568 1.132 0.715 0.715

RIi 0.475 0.122 3.885 0.000 0.235 0.715 0.656 0.656

RIs ~~

RIi 0.435 0.079 5.500 0.000 0.280 0.590 0.621 0.621

Intercepts:

Estimate Std.Err z-value P(>|z|) ci.lower ci.upper Std.lv Std.all

.c1mean2 3.644 0.093 39.387 0.000 3.462 3.825 3.644 2.482

.c2mean2 3.565 0.094 37.751 0.000 3.380 3.750 3.565 2.322

.c3mean2 3.590 0.092 39.116 0.000 3.410 3.769 3.590 2.380

.s1mean 3.697 0.068 54.632 0.000 3.564 3.829 3.697 2.850

.s2mean 3.677 0.088 42.012 0.000 3.505 3.849 3.677 2.789

.s3mean 3.764 0.085 44.431 0.000 3.598 3.930 3.764 2.851

.i1mean6 4.469 0.046 97.821 0.000 4.379 4.558 4.469 5.109

.i2mean6 4.475 0.038 116.368 0.000 4.400 4.551 4.475 5.547

.i3mean6 4.388 0.053 82.500 0.000 4.284 4.492 4.388 5.171

RIc 0.000 0.000 0.000 0.000 0.000

RIs 0.000 0.000 0.000 0.000 0.000

RIi 0.000 0.000 0.000 0.000 0.000

wc1 0.000 0.000 0.000 0.000 0.000

.wc2 0.000 0.000 0.000 0.000 0.000

.wc3 0.000 0.000 0.000 0.000 0.000

ws1 0.000 0.000 0.000 0.000 0.000

.ws2 0.000 0.000 0.000 0.000 0.000

.ws3 0.000 0.000 0.000 0.000 0.000

wi1 0.000 0.000 0.000 0.000 0.000

.wi2 0.000 0.000 0.000 0.000 0.000

.wi3 0.000 0.000 0.000 0.000 0.000

Variances:

Estimate Std.Err z-value P(>|z|) ci.lower ci.upper Std.lv Std.all

.c1mean2 0.000 0.000 0.000 0.000 0.000

.s1mean 0.000 0.000 0.000 0.000 0.000

.i1mean6 0.000 0.000 0.000 0.000 0.000

.c2mean2 0.000 0.000 0.000 0.000 0.000

.s2mean 0.000 0.000 0.000 0.000 0.000

.i2mean6 0.000 0.000 0.000 0.000 0.000

.c3mean2 0.000 0.000 0.000 0.000 0.000

.s3mean 0.000 0.000 0.000 0.000 0.000

.i3mean6 0.000 0.000 0.000 0.000 0.000

RIc 1.229 0.236 5.210 0.000 0.767 1.692 1.000 1.000

RIs 1.150 0.112 10.296 0.000 0.931 1.369 1.000 1.000

RIi 0.427 0.076 5.584 0.000 0.277 0.577 1.000 1.000

wc1 0.926 0.225 4.105 0.000 0.484 1.368 1.000 1.000

.wc2 0.783 0.119 6.590 0.000 0.550 1.015 0.694 0.694

.wc3 0.660 0.084 7.846 0.000 0.495 0.825 0.631 0.631

ws1 0.533 0.101 5.292 0.000 0.335 0.730 1.000 1.000

.ws2 0.468 0.097 4.829 0.000 0.278 0.658 0.796 0.796

.ws3 0.465 0.077 6.021 0.000 0.314 0.616 0.784 0.784

wi1 0.338 0.083 4.056 0.000 0.175 0.502 1.000 1.000

.wi2 0.194 0.066 2.966 0.003 0.066 0.323 0.868 0.868

.wi3 0.260 0.046 5.679 0.000 0.170 0.349 0.886 0.886

Defined Parameters:

Estimate Std.Err z-value P(>|z|) ci.lower ci.upper Std.lv Std.all

sa1 0.059 0.048 1.228 0.219 -0.035 0.154 0.050 0.050

sa2 0.025 0.030 0.841 0.401 -0.034 0.085 0.017 0.017

sa3 0.082 0.043 1.910 0.056 -0.002 0.166 0.051 0.051

totalsc 0.167 0.080 2.075 0.038 0.009 0.324 0.118 0.118

ia1 0.059 0.051 1.163 0.245 -0.040 0.158 0.031 0.031

ia2 0.025 0.062 0.411 0.681 -0.096 0.147 0.017 0.017

ia3 0.134 0.069 1.945 0.052 -0.001 0.268 0.066 0.066

totalic 0.218 0.148 1.478 0.139 -0.071 0.507 0.114 0.114
